# Active and latent tuberculosis among inmates in La Esperanza prison in Guaduas, Colombia

**DOI:** 10.1371/journal.pone.0209895

**Published:** 2019-01-25

**Authors:** Julio Guerra, Daniel Mogollón, Deccy González, Ricardo Sanchez, Zulma Vanessa Rueda, Carlos A. Parra-López, Martha Isabel Murcia

**Affiliations:** 1 Grupo MICOBAC-UN, Departamento de Microbiología, Facultad de Medicina, Universidad Nacional de Colombia, Bogotá DC, Colombia; 2 Programa de Tuberculosis y Lepra, Secretaría de Salud de Cundinamarca, Bogotá DC, Colombia; 3 Departamento de Psiquiatría, Facultad de Medicina, Universidad Nacional de Colombia, Bogotá DC, Colombia; 4 Facultad de Medicina, Universidad Pontificia Bolivariana, Medellín, Antioquia, Colombia; Jamia Hamdard, INDIA

## Abstract

**Introduction:**

Active tuberculosis (TB) and latent tuberculosis infection (LTBI) are a public health threat in prisons around the world. The objectives of the study were to estimate the prevalence of LTBI and TB as well as to investigate TB transmission inside one prison, in Colombia.

**Methods:**

A Cross-sectional study was conducted in inmates who agreed to participate. Inmates with respiratory symptoms (RS) of any duration underwent to medical evaluation and three sputum samples were taken for smear microscopy and culture for TB diagnosis. Drug susceptibility was analyzed using BACTEC MGIT 960 and GenoType MTBDRplus. Molecular genotyping of *Mycobacterium tuberculosis* isolates was performed by 24-Locus MIRU-VNTR and spoligotyping. LTBI was evaluated according to the result of the tuberculin skin test (TST). Close contact investigation was conducted inside the prison for inmates that shared the cell with the index TB case.

**Results:**

Among 301/2,020 (15%) inmates with RS of any duration, 8% were diagnosed with active TB. The prevalence of active TB was 1,026 cases/100,000 inmates. We isolated *M*. *tuberculosis* in 19/24 (79%) TB cases, 94.7% were susceptible to first line drugs and only one was monoresistant to isoniazid. The most prevalent sub-lineage was Haarlem (68.4%), followed by LAM (26.3%) and T superfamily (5.3%). 24-Locus MIRU-VNTR typing results alone or in combination with spoligotyping identified three clusters containing two isolates each. Two clusters corresponded to inmates that shared the same cell, but each one was located in different blocks of the prison. Inmates from the last cluster were in the same block in nearby cells. TST reading was performed in 95.6% inmates, and 67.6% had a positive reaction.

**Conclusions:**

The prevalence of LTBI and TB was higher in prison than in the general population. Molecular genotyping suggests that TB in this prison is mainly caused by strains imported by inmates or endogenous reactivation.

## Introduction

Tuberculosis (TB) in prison population is an important public health problem, especially in low and middle and income countries [1]. Regardless of the economic status and the TB burden of the country, the estimated prevalence of latent TB infection (LTBI) and active TB in prison are reported to be higher than in the general population [1–3]. Colombia is not an exception, studies of incidence and prevalence of TB in prisons have reported values that are higher than those found in the general population [4–7]. In addition, it is important to notice that Colombian prison population trend almost duplicated the number inmates from 60,021 in 2006 to 118,532 inmates in 2016 [8].

Several risk factors contribute to higher incidence of TB in prisons. Some factors are related to characteristics of the prison population itself and others are attributable to conditions of incarceration such as overcrowding. Other factors are associated to problems in TB control programs like the implementation of measures to control TB infection and limited access to adequate health care services in prison´s settings [3, 9]. In addition, inmates may be at risk of rapid progression from LTBI to active TB due to co-morbidities, such as HIV infection [9].

Diagnosis of active TB is very important for the control and prevention of the disease. Mostly, TB screening is conducted when a person presents persistent cough for more than two weeks, accompanied or not by other respiratory and/or constitutional symptoms [5, 10]. However, different studies have shown low sensitivity and specificity of this diagnostic criterion of TB in HIV infected patients [11–13]. In addition, one study conducted in four Colombia prisons found that 25% of cases had less than 15 days of respiratory symptoms (despite no immunosuppression). This information highlight the importance of expanding the World Health Organization (WHO) criteria to other high-risk population groups such as inmates [5].

Diagnosis and treatment of LTBI can reduce the risk of development of active disease, especially in high-risk groups of progression to active TB. There are two approved methods for the detection of LTBI, the tuberculin skin test (TST) and the interferon-gamma release assays (IGRA) [2, 14]. In Colombia, a national guideline published in 2015, recommend diagnoses of LTBI through TST and use of IGRA in few particular cases [15]. According to this guideline, there are some priority groups for the diagnosis of LTBI such as HIV infected patients, children in contact with a TB case, people under biological immunomodulator therapy for autoimmune diseases, in dialysis, or that is going to receive hematopoietic stem cell or solid-organ transplants and people with silicosis. However, this guideline did not include the diagnosis of LTBI in inmates [15].

The Center for Diseases Control and Prevention (CDC) use spoligotyping and 24-locus based MIRU-VNTR typing methods for TB genotyping of all *M*. *tuberculosis* isolates running at the US National TB Genotyping service (NTGS). TB genotyping data, when combined with epidemiologic information analyses, contributes to the identification of individuals with TB involved in a chain of recent transmission and distinguish recent infection (with development of active disease) from reactivation. In addition, it identifies individuals with TB disease involved in an outbreak [16]. Contact investigation is not only important for prevention of future cases [17] but also it might be more cost-effective when the number of patients is small rather than after a large outbreak has established [18], especially in high risk population groups such as inmates due to the presence of numerous TB risk factors.

Based on the aforementioned, the aims of our study were to estimate the prevalence of LTBI and active TB as well as to investigate TB transmission inside one prison.

## Material and methods

### Study design and setting

A cross-sectional study was conducted during three weeks (September–October, 2015) in La Esperanza prison, located in the rural area of the municipality of Guaduas in Colombia. This prison has three levels of security (maximum, medium and minimum) and is exclusively for adult males. The maximum prison capacity is 2.824 inmates. The prison population held during the study period was 2.630 inmates.

### Eligibility criteria

Inmates older than 18 years of age, who freely accepted to participate and to sign a written consent form, were included in the present study. Individuals that did not accept to participate in the study or those that accepted but did not sign the informed consent form were not included.

### Data collection

A general structured questionnaire was used to collect information from all prisoners (S1 and S2 questionnaires). The questionnaire included: age; ethnic group; nationality; education level; history of previous prison sentences; time and history of the current incarceration; history of prior TB (including date of last episode, treatment and outcome); previous contact with TB cases (outside and/or inside the prison); HIV status; number of inmates in same cell; and the presence of cough or expectoration of any duration.

### Clinical evaluation

A physician registered the clinical history and conducted a physical examination to all inmates that reported respiratory symptoms of any duration. Specific query questions included: comorbidities (cancer, transplants, diabetes, rheumatoid arthritis, chronic obstructive pulmonary disease, chronic kidney disease, and any other immunosuppressive disease); use of drugs (inhaled, injected, or smoked) or alcohol, along with the quantity and time of consumption; fever; weight loss; night sweats; hemoptysis; BCG scar; weight and height.

### Detection of latent TB infection (LTBI)

After the interview, trained nurses administered to inmates the tuberculin skin test (TST) (0.1 ml of purified protein derivate, PPD RT23–2 tuberculin units; Staten Serum Institute, Copenhagen, Denmark), following recommendations from US Center for Disease Control and Prevention (CDC) recommendations. The same nurse who injected PPD read the reaction 72 hours later and recorded the diameter of induration in millimeters. A TST was considered positive if the induration was ≥10 mm for immunocompetent persons and ≥ 5 mm for HIV positive patients [19].

### Diagnosis of active TB

Sputum samples from inmates with RS of any duration were subject to sputum smear microscopic analyses and culture. In brief, three sputum samples were collected on consecutive days by specialized team personnel either by spontaneous sputum collection or induced sputum in cases were an inmate was not able to collect the sputum spontaneously. Both procedures were performed following protocols and biosafety measures described by Rueda et., al 2015 [20]. Sputum samples were transported and maintained at 4°C until processing in the laboratory of Mycobacteriology (UN-MICOBAC) at the Universidad Nacional de Colombia, Bogota, DC. Colombia. Sputum samples were processed by decontamination with *N*acetyl-L-cysteine-sodium hydroxide method and the concentrated sediments were subjected to smear microscopy using the Ziehl–Neelsen (ZN) staining method [21]. Furthermore, the concentrated sediments of the first sample of each patient was cultured per duplicate on Lowenstein-Jensen (L/J) culture media and into one mycobacterial growth indicator tube (MGIT) incubated in MGIT 460 BACTEC instrument (BD Diagnostics, Sparks, MD, USA). All clinical isolates obtained from culture (solid and/or liquid), were identified as members of the *Mycobacterium tuberculosis* complex (MTBC) by using the BD MGITTBc Identification Test (TBc ID) according to the manufacturer’s instructions.

A pulmonary TB case was defined as a positive result in the smear microscopy by ZN staining method indicating the presence of Acid-Fast Bacilli (AFB) in the sputum. A negative result indicates that no acid-fast bacilli were seen in 100 fields and the individual tested was considered to have no smear positive pulmonary TB [22]. Furthermore, a patient with *Mycobacterium tuberculosis* complex identified from a sputum specimen by culture was considered a pulmonary TB case as well [5].

### HIV testing for active TB cases

An immunoassay for qualitative detection of HIV-1/HIV-2 antibodies in human blood test was conducted for all TB cases diagnosed in the present study using HIV 1.2.O Rapid Test Cassette test (Whole Blood/Serum/Plasma) (*Screen*Italia) following the manufacturer recommendations [23]. If a positive result was obtained in the first test, a confirmatory ELISA test was conducted in a reference hospital. HIV treatment, patient education and counselling support services was provided to HIV-positive individuals.

### Contact tracing investigation

Contact-tracing investigation inside the prison was conducted during the study as soon as an inmate was diagnosed with active TB. First, we identified inmates who shared a cell with a TB case and assigned them as high-priority contact if they have been together for more than a week. In general inmates are confined in cells for more than 13 hours per day, an exposure time considered to be high. Second, all high priority contacts were evaluated to rule out active TB according to the procedures described above.

### Drug susceptibility testing (DST)

*M*. *tuberculosis* isolates were subject to first line drug susceptibility testing using BACTEC MGIT 960 SIRE kit [Becton Dickinson, CA, USA] but only for isoniazid (INH) and rifampicin (RMP) following the manufacturer’s instructions [24]. In addition, GenoType MTBDR*plus* testing was conducted blinded from the phenotypic DST results according to the manufacturer's recommendations (http://www.hainlifescience.de).

### TB genotyping and molecular data analysis

Genomic DNA was isolated from positive *M*. *tuberculosis* culture using the PureLinkGenomic DNA Mini Kit (Catalogue number K1820-01- Invitrogen) following the instruction of the manufacturer and quantified by using NanoDrop system. Spoligotyping molecular typing method was carried out using the commercially available membranes (Ocimum Biosolutions, Hyderabad, India) [25]. Standard 24-locus based MIRU-VNTR typing method was performed for all isolates using the methodology described by Supply *et al*., 2006. Briefly, the PCR products were separated onto a 2% agarose ethidium bromide-stained gel, and DNA bands were visualized and recorded under ultraviolet light using the Chemi Genius 2 Bio Imaging System (Syngene). Molecular weight of each fragment were determined using GeneSnap software Version 6.07 GeneTools (Syngene), and the corresponding repeat number was determined by using standard allelic table described by Supply *et al*., 2006.

Spoligotyping results were converted into octal codes and entered in SITVIT database to determine the spoligotyping lineage, sub-lineage and family distribution [26]. Phylogenetic lineage identification was performed by using online tools available from MIRU-VNTRplus website (www.miruvntrplus.org) [27]. Two patients were considered to have matching genotypes if their isolates had the same spoligotype and/or MIRU patterns. A genotype cluster was defined as two or more TB patients with matching genotype [16]. Molecular clustering analysis was determined by constructing a dendogram based on spoligotyping and MIRU-VNTR data.

TB genotyping results were combined with epidemiologic data analyses to establish if TB patients of the same genotype cluster (same spoligotype and MIRU patterns) were involved in the same chain of recent transmission. Information on epidemiologic links among TB patients from the same cluster were collected from the questionnaires mentioned above. Two patients said to have a known epidemiologic link if at least one of the following conditions apply. *i)* One of the patients named the other as a contact during one of the patient’s infectious period or *ii)* the two patients were at the same place at the same time during one of the patient’s infectious period. By definition, the infectious period for a sputum smear-positive case, extend from three months before the first positive smear or symptom onset; until two weeks after the time of the start of TB treatment or until the patient is placed into isolation or the date of the first negative smear that is followed by consistently negative smears. The beginning of the infectious period for a sputum smear-negative case was defined as one month before the onset of symptoms, the TB treatment began, the patient was placed into isolation; or two weeks after the TB treatment started or until isolation [18].

### Statistical analysis

Epidemiological data were entered in Microsoft Access database. Discrepancies were checked against the crude data, inconsistencies were confirmed and missing data were collected again by personnel of the field team. Ten percent of questionnaires were randomly selected to ensure the quality and completeness of information. The database is attached as S1 Dataset.

The LTBI prevalence was calculated using as numerator the number of inmates with a PPD positive and as a denominator, the number of inmates in which TST was administered and read. The active TB prevalence rate was estimated using as numerator the number of TB cases diagnoses either by sputum smear and/or by culture plus the TB cases who were in TB treatment in the prison; and the denominator was the total number of inmates who were held in the prison. For both we estimated the point prevalence due to we were for three weeks.

Univariate analysis for baseline characteristics was performed using absolute and relative frequencies for quantitative variables. The age, time of incarceration and time with cough were reported as median with their interquartile ranges. We did a bivariate analysis to identify potential risk factors associated TB, and considered significant a p-value <0.05. Comparisons between medians and percentages were performed using Wilcoxon rank-sum test and Fisher’s exact test respectively. Statistical analysis was performed using STATA 11.1 (StataCorp, College Station, TX, USA). We could not do a multivariate analysis due to the low number of TB cases, and therefore it is not adequate to run it and to draw conclusions about risk factors.

### Ethical approvals and considerations

This study was approved by the ethics committee of the School of Medicine (Universidad Nacional de Colombia), the governmental institution responsible for the administration and security of the national prison system (Instituto Nacional Penitenciario y Carcelario–INPEC); and by the prison were the study was conducted. Additionally, prisoners who accepted to participate voluntary in the present study signed a consent form that was previously explained by the field team. It is important to notice that there were separate consent forms for TB and HIV.

Those inmates that were not enrolled in the present study, but complained of respiratory symptoms or were positive for any pathology or medical condition during the clinical evaluation by the field team (physicians), were reported to the health care service at the prison in order to be treated. We also notified inmates diagnosed with TB and/or HIV to facilitate the initiation of the corresponding treatment and assistance. TB treatment was provided and monitored by the state health department (Secretaria de Salud de Cundinamarca). Inmates diagnosed with active TB were isolated in individual cells in order to prevent disease transmission.

To keep personal health information (PHI) confidential, all documents were protected by coding and password, and only some staff involved in the study had access to this data. The study team was absolutely prohibited from sharing information with people not related with the research.

## Results

### Prevalence of LTBI and active TB

**Fig 1:**
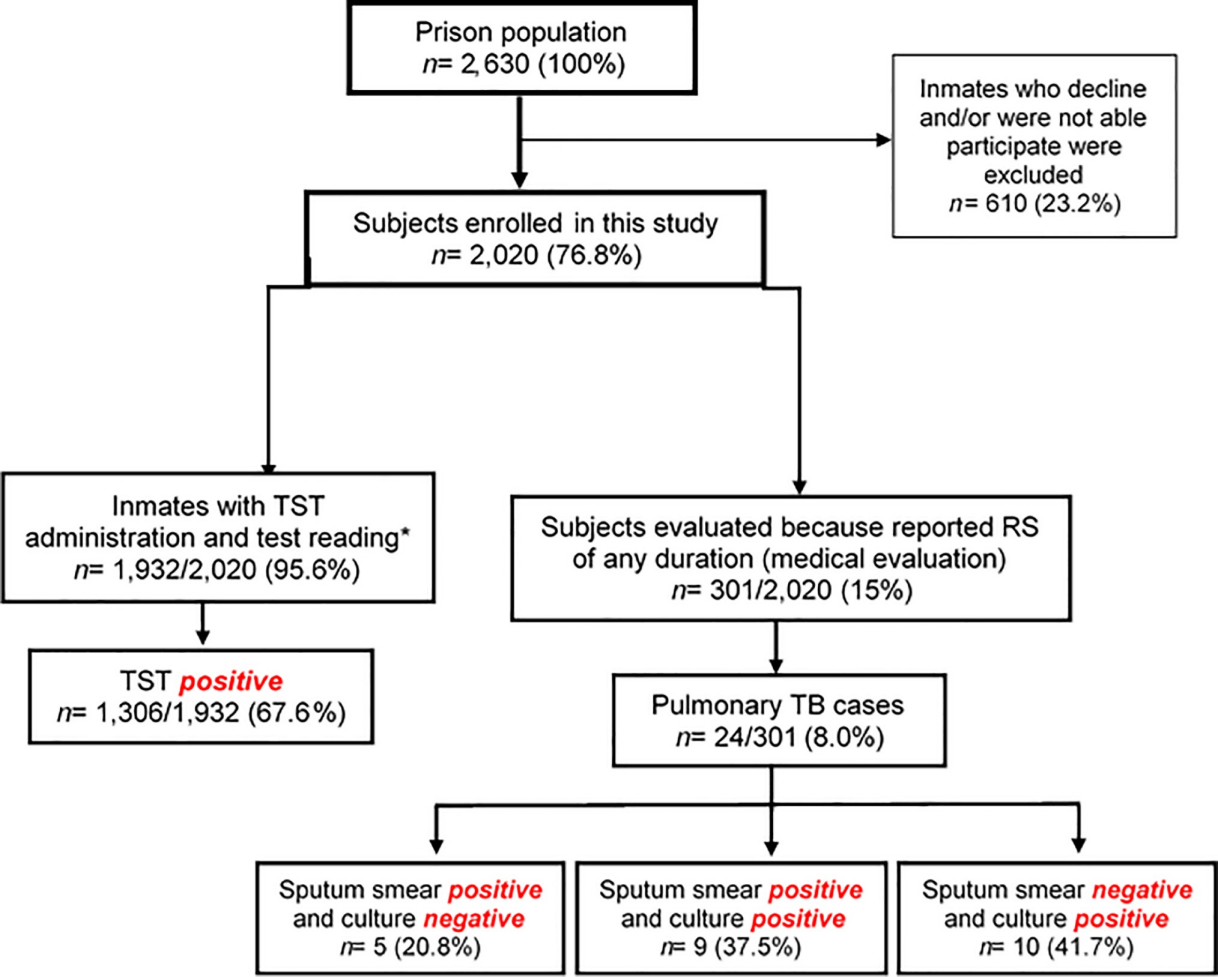
Flowchart of inmates included in the study. *Refusal to participate, reading could not be performed and/or some inmates were involved in prison routine activities or outside permission.

From the total prison population, 76.8% (2,020/2,630) were screened using a structured questionnaire. TST was performed and read in 1,932/2,020 (95.6%), and in 1,306 (67.6%) of them TST was positive. Regarding to TB, 301/2,020 (15%) subjects were evaluated because they reported RS of any duration, and 24/301 (8.0%) of them were diagnosed with active TB (Fig 1). According to the DST results and following DOTS Colombian guidelines, the 24 new cases received anti-TB treatment and all of them responded well to therapy. When the study begun, three inmates were already isolated and under TB treatment, for a total of 27 TB cases within the prison. The overall prevalence of active TB was at the prison 1,026 cases /100,000 inmates (27/2630). Regarding to the contacts, none of the inmates that share the cell with a TB case (high priority contact) was diagnosed with active TB during the study.

### Univariate analysis of inmates with positive and negative TST results

The median age of inmates with a TST positive was 29 years and the median time of incarceration was 30 months. In addition, history of prior incarceration and a prior contact with a TB case was reported in 25% and 36.5% of inmates with a positive TST, respectively. Finally, all HIV infected patients were PPD positive (Table 1). The median size of induration among non-HIV inmates, including the TB cases was 10 (ranges 10–30) and among HIV patients was 11 (ranges 10–12). Statistical analysis of TST reaction showed that only age and no contact with a TB case had significant differences.

**Table 1:** Baseline characteristics of inmates with positive and negative TST results.

CHARACTERISTICS		PPD positive (n = 1306)	PPD negative (n = 626)
	***median (ranges)***		***median (ranges)***
Age (years)		29 (19–73)	30 (19–76)*
Time of incarceration (months)		30 (1–213)	32 (4–220)
		**Frequency (%)**	**Frequency (%)**
History of prior incarceration		330 (25.7)	146 (23.3)
HIV positive status		6 (1.4)	0.0 (0.0)
No contact with a TB case		831 (63.6)	441 (70.4)**
Contact with a TB case inside the prison		448 (34.3)	177 (28.3)
Contact with a TB case outside the prison		22 (1.7)	5 (0.8)
Contact with a TB case inside and outside the prison		5 (0.4)	3 (0.5)

*Two-sample Wilcoxon rank-sum, p = 0.04

** Fisher’s exact test, p = 0.003

### Univariate analysis of inmates with respiratory symptoms of any duration

The total number of inmates that reported respiratory symptoms (RS) of any duration (*n* = 301) was divided in two groups, inmates with and without TB (Table 2). The median age of 26 years was the same in both groups. Despite the median time of cough was the same in both groups, 10/24 (41.7%) of TB cases had less than 15 days of cough. Among those cases, 4/10 (40%) were sputum smear positive. 8/24 (33.3%) of TB cases presented abnormal breath sounds on lung auscultation. In the group of TB cases, 11/24 (45.8%) reported a contact with a TB case in the past, and they all said the contact was while in prison. Just 1/24 (4.2%) of the TB cases had a history of prior TB, who completed treatment in the same prison and the according to the record outcome was cured. This person had been in the prison for the last 24 months, which means that he got sick with TB in the same prison both times. Regarding to the HIV status, 1/24 (4.2%) cases of TB had prior diagnoses of HIV; and the others 23/24 TB cases were HIV negative (Table 2). Statistical analysis showed that not significant differences were found, except for abnormal breath sounds on lung auscultation.

**Table 2:** Baseline characteristics of inmates with respiratory symptoms of any duration.

CHARACTERISTICS	TB cases (n = 24)	Non-TB cases (n = 277)
	median (ranges)	median (ranges)
Age (years)	26 (22–72)	29 (19–70)
Time of incarceration (months)	35 (13–94)	30 (5–131)
Time with cough (days)	15 (4–90)	15 (1–90)
	Frequency (%)	Frequency (%)
Respiratory symptoms (<15 days)	10 (41.7)	129 (46.6)
Abnormal breath sounds on lung auscultation*	8 (33.3)	30 (10.8)
History of prior incarceration	9 (37.5)	84 (30.3)
Contact with a TB case	11 (45.8)	154 (55.6)
History of prior TB	1 (4.2)	13 (4.7)
HIV positive status	1 (4.2)	3 (1.1)
Current drug use	15 (62.5)	191 (69.0)
Smoked (cigarettes)	10 (41.7)	153 (55.2)
Co-morbidities	1 (4.2)	15 (5.4)
Cough	24 (100)	277 (100)
Expectoration	22 (91.7)	218 (78.7)
Fever	5 (20.8)	83 (30.0)
Weight loss	12 (50)	121 (43.7)
Hemoptysis	2 (8.3)	23 (8.3)
BCG scar	21 (87.5)	226 (81.6)
Body Mass Index		
Normal (18–25 kg/mt2)	21 (87.5)	228 (82.3)
Underweight (<18 kg/mt2)	0 (0.0)	1 (0.4)
Overweight (>25 kg/mt2)	3 (12.5)	48 (17.3)
Location in the prison (block)**		
Block A	4 (16.7)	25 (9.0)
Block B	2 (8.3)	26 (9.4)
Block C	0 (0.0)	32 (11.6)
Block D	0 (0.0)	24 (8.7)
Block E	1 (4.2)	33 (11.9)
Block F	4 (16.7)	32 (11.6)
Block G	3 (12.5)	31 (11.2)
Block H	3 (12.5)	17 (6.1)
Block I	2 (8.3)	9 (3.2)
Block J	2 (8.3)	20 (7.2)
Block K	3 (12.5)	28 (10.1)

* Fisher’s exact test, p = 0.005

** Block identification was changed for security reasons

### Drug susceptibility

DST data was available for 19/24 (79%) of TB cases because we were able to recover 19 isolates. DST results showed that 18/19 (94.7%) of the strains were susceptible to all drugs, while only 1/19 (5.3%) was mono-resistant to INH by both methods used (Table 3).

**Table 3:** Drug Susceptibility Test (DST) results.

	Patients isolates (n = 19)				
Method	Pan-susceptible	Mono RIF	Mono INH	MDR	Total
BACTEC MGIT 960 SIRE kit	18	0	1*	0	19
GenoType MTBDR plus	18	0	1*	0	19

*The same strain was mono-resistant by both methods. RIF: Rifampicin. INH: Isoniazid. MDR: Multi-drug resistant TB.

### Molecular data analysis

Spoligotyping results showed that all *M*. *tuberculosis* isolates belonged to the Euro American lineage and were classified within seven SITs numbers according to the international SITVIT database (SIT 33, 42, 47, 50, 53, 62, and 727). 16/19 (84.2%) isolates were found to be grouped into four clusters, containing two to eight isolates per cluster. The distribution of the spoligotype sub-lineage observed in this study were in order: Haarlem, 13/19 (68.4%) with the following family distribution: H3 with 8/13 (61.5%) and H1 with 5/13 (48.5%). Latin-American & Mediterranean (LAM), 5/19 (26.3%) with the following family distribution: LAM9 with 4/5 (80%) and LAM3 with 1/5 (20%). Finally, T superfamily 1/19 (5.3%) with the presence of T1.

**Fig 2:**
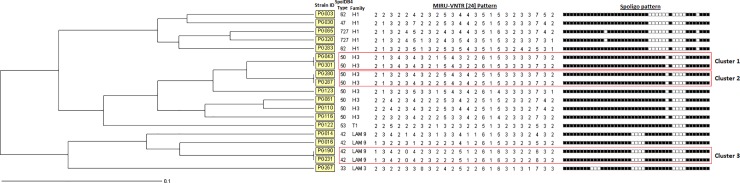
Results obtained, including genotyping information by spoligotyping and 24-loci MIRU. UPGMA-Tree, MIRUS-VNTR[24]: categorical (1), Spoligo: Categorical (1) *Clusters are labeled in red boxes.

Molecular cluster analysis based on 24-locus MIRU-VNTR genotype in conjunction with spoligotyping results showed that 6/19 (31.6%) isolates were found to be grouped into three clusters, containing two isolates each; and 13/19 (68.4%) isolates present a unique type (Fig 2). Epidemiological information analysis of each cluster showed that they were detected in different blocks of the prison. TB patients from cluster 1 as well as cluster 2 were sharing the same cell for three and eight weeks, respectively. TB patients from cluster 3 did not share the same cell but they all were incarcerated in the same block in nearby cells, and they declared to know each other. One patient of each cluster was sputum smear positive. Any patient of these clusters were HIV positive or have another comorbidity but illicit drug use, smoking and lost weight were common among them.

## Discussion

The prevalence of LTB infection in this prison (67.6%) was higher than the estimated for the Americas general population (25%) [28], including Colombia [29]. The prevalence of TST positivity was similar to previous studies in three Brazilian prisons: 61.5% [30], 64.1% [31], and 73% [32]; and Malaysia, 88.8% [33]. In Colombia, a recent study reported a high prevalence of LTBI (77%) (first TST positive (66%) followed by a second TST positive (11%) in original negative inmates); which indicate the importance of the second TST [7]. In our study, we did not perform a second TST for inmates with a negative TST result in the first administration (32.9%), which may increase the overall prevalence of LTBI in the present study. In contrast, our study reported prevalence of LTBI in La Esperanza prison higher than other studies in prisons of some low/middle income countries, where the TST prevalence was 15–33% and 49% for Brazil [34, 35], 48% in Pakistan [36], and 52.4% in Nigeria [37].

The prevalence of LTB infection may be influenced by a prior BCG vaccination, which is known to cause false-positive TST results. However, two meta-analysis showed that age at vaccination is an important modifier in the effect of BCG vaccination on TST reactivity. The effect on TST of BCG received during infancy is minimal, especially after ≥10 years post-vaccination [38, 39]. Moreover, BCG vaccination after infancy was associated with an increased risk of TST reactivity in the first 15 years after vaccination [38]. It is important to notice that in Colombia, BCG vaccine is administered at the time of birth [40, 41] and the national vaccine coverage is around 90% according to WHO vaccine-preventable diseases monitoring system [42]. In our study, all inmates were ≥18 years of age and because of the age of BCG vaccination in Colombia and the above explanation effect of BCG vaccination on TST, let us to argue that the high prevalence of LTB infection in this study may not be influenced by BCG vaccination.

In Colombia LTBI treatment with isoniazid is not recommended for immunocompetent persons with a positive TST result, however the importance for clinical follow-up should be highlighted, especially in HIV-infected people which are known to be in high risk to become active TB [7, 9]. In our study, four HIV-infected patients with a positive TST result were notified to the prison health office in order to study for active TB. A recent study conducted in Colombia, reported an incidence of LTBI during two years of follow-up of negative TST prisoners at baseline of 29.5%, which highlights the importance to follow-up those negative TST prisoners [43] with a number needed to screen of 3.4 people to detect one positive converter among those negative TST at baseline.

In the present study, the prevalence of active TB was 1,026 cases per 100,000, value 38.3 times higher than the Colombian general population (26.8 cases per 100,000) as reported for 2017 [44]. This is similar to the literature that has reported to be higher than the general population, regardless to the economic status and the population TB burden of the country [3]. For instance, in prisons from Ethiopia (3 times), Turkey (4 times), Bangladesh (4 times), South Africa (9 times), and Zambia (18 times) are higher than the general population, respectively [22, 45–48].

In South America, Brazil reports a prevalence of active TB in inmates that range between 21.4 to 80 times higher than the Brazilian general population [31, 32, 49, 50]. In Colombia, a study conducted in a prison in the capital (Bogota DC) in 2010, reported a prevalence that was 3.8 times higher than in the Colombian general population [4]; which was substantially lower than the reported in the present study. Another study, in 2010 and 2011 described a TB incidence among inmates was 3.18 and 4.5 fold higher than it was within the general population [6]. A recent study related with the incidence of active TB and conducted in four different prisons in Colombia reported an estimate incidence, which was 20 times higher than the general population. The high prevalence of active TB found by Rueda and in our study *et al*. [5] may be explained by the change of the respiratory symptoms criteria of 15 days or more for any duration, and the combined use of methods, including the MGIT liquid culture.

Regarding to the duration of symptoms, Rueda *et al*., 2013 founded that 25% of 72 cases had less than 15 days of respiratory symptoms and 66.7% of them were sputum smear positive and probably infectious [5]. In our study, we found that 41.7% of the TB cases were people with respiratory symptoms with a duration of less than 15 days, and 4 (40%) of them were sputum smear positive. In contrast, the WHO screening algorithm recommends that all people with cough lasting longer than 2 weeks should be investigated for TB in immunocompetent people [51]. If we had followed this recommendation, we could not be capable to detect 41.7% of the TB cases.

In addition, another important criterion for the diagnosis of TB in prisons is the importance of using sputum culture. In the present study, 41.7% of TB cases were detected only by culture. Similar results were obtained in a study conducted in four Colombian prisons, were 23.6% of 72 cases were sputum smear negative and culture positive. Despite these results, this diagnostic method is not well implemented in the Colombian prison system; regardless of the national and international recommendations [5]. In our study, just one case of confection HIV/TB was detected; however, there were three additional HIV infected patients in the prison that should be monitored because all of them had a positive TST result and probably they are in a higher risk of rapid progression to active TB.

DST results showed that only one isolated was mono-resistant to isoniazid and no cases of MDR-TB were detected. A retrospective study conducted in Colombia, in which they analyzed the susceptibility profile of 72 isolates obtained from inmates from different prisons in Colombia reported that two TB cases were mono-resistant to isoniazid and two were MDR-TB; the latter two were previously treated cases [52]. Other study conducted in four Colombian prisons that evaluate the susceptibility profile of 72 isolates, reported just one TB case was mono-resistant to isoniazid and no MDR-TB cases were founded [5].

Spoligotyping data analysis showed that sub-lineages Haarlem (68.4%) and LAM (26.3%) were the most prevalent, accounting for 94.7% of all strains. Similar results were reported in Latin American countries, although LAM is the most prevalent reported sub-lineage in most countries. Brazil (LAM: 53.7% y Haarlem: 7%); Venezuela (LAM: 53%, Haarlem: 5%); and Peru (LAM: 28.3%, Haarlem: 28%), respectively [53–55].

In Colombia, two recent studies which evaluated strains from different regions of the country reported a predominance of those two sub-lineages as well: (LAM, 39.9% and Haarlem, 19%) and (Haarlem, 44.3% and LAM, 38.5%), respectively [56, 57]. However, in the second study conducted in Medellin, Cali and Popayan cities, Haarlem was the most common as we reported in our study. Regarding to the sub-lineage distribution in prison population, a study conducted in Brazil found that LAM (40%) was the most common followed by T superfamily (22%) and Haarlem (17.5%) [50]. One study conducted in four Colombian prisons (Medellin and Bucaramanga cities) reported LAM and Haarlem were the most prevalent sub-lineages (LAM: 56.8% and Haarlem: 36.4%) [58] as we reported here.

A combination of spoligotyping and 24-loci MIRUs has been successfully used in resources- limited settings to predict “potential” transmission chains [59–61]. In addition, clustering reflecting recent and active transmission of TB depends on study duration [62]. We were unable to identify the index TB case and the secondary case in each cluster because the study’s short time duration. However, the presence of known epidemiological links in each cluster, the prolonged time of exposure and the fact that one patient of each cluster was sputum smear positive and presumably highly infectious to other [18] could be a strong suggestion of recent transmission among inmates of these three clusters. The low proportion of clustered isolates (31.5%) in La Esperanza prison may indicate that TB in this prison was mainly caused by strains imported by inmates or endogenous reactivation [62, 63].

The overall goal of TB contact investigation is to halt the TB transmission of *M*. *tuberculosis* by the identification, isolation and treatment of a person with active TB; and to identify contacts with active TB or LTBI [17, 18, 64]. In prison settings, this approach should be undertaken promptly after an inmate with active TB has been identified [64]. Despite the investigation with contacts showed that none of the high-priority contacts (inmate that share the cell with a TB case) were diagnosed with active TB, interestingly, 46% of TB cases in the present study reported had been in contact only with TB cases while they have been in prison. It is important to mention that this is a cross-sectional study that does not allow us to conclude about the impact of contact sharing with an index case in prison, because that needs a cohort study for at least two years after the contact with the TB case or the TST conversion to evaluate TB transmission.

In addition, contact investigation will prevent new cases and it might be more cost-effective when the number of patients is small rather than after a large outbreak has established [17, 18]. In our study, we found that 6 out of 24 TB cases detected were in a cluster and had an epidemiological link. A published retrospective cohort analyses of TB genotype clusters showed that the two most important factors that predicted outbreaks were the presence of at least one patient who reported homelessness, excess alcohol use, illicit drugs use, or incarceration, and rapid initial cluster growth. That study also suggest that if recent transmission of TB occurs among patients with the above-described social risk factors, the risk of a TB outbreak increases [18]. In prisons, several papers have shown the importance of contact tracing in this environment due to the higher risk of LTBI among contacts of index cases [65, 66] and the high numbers of TB cases detected when active case finding program is implemented [67].

Among TB cases, just one of them had a history of prior TB and its last episode was just 10 months. Despite this patient completed his last treatment and the outcome was cure. We could not determine if the second episode of a TB patient diagnosed in the present study was due to a reactivation or an exogenous re-infection after curative treatment. We did not have the isolate from the first episode in order to genotype and compare the patterns. This is important because this person has been in the prison for the last 24 months, which means that he was sick with TB in the same prison both times. One explanation for this relapse could be that he had cavities that we could no detect because in prisons there is limited access to chest x rays, or he became infected during his incarceration.

One of the most important limitations of this study was that during the screening of the LTBI, we did not perform the two-steps TST when the initial TST was negative. Certain individuals with *M*. *tuberculosis* infection will have a negative TST when tested many years after the initial infection. The initial skin test, probably stimulate or “boost” to the immune system’s ability to react to tuberculin and cause a positive reaction to subsequent tests [14].

Another important limitation was that we did not performed radiography-based screening. The accomplishment of this screening in a health institution outside the prison was not possible due to security matters and budget. Because of the importance of early TB diagnosis in setting such as prisons, chest x-ray could help in the detection of active TB cases in the prison, and probably we could underestimate the active TB prevalence.

Regarding contact investigation activities, we could not evaluate other contacts inside the prison such as inmates in the same block. It can be explained by the fact that the number of inmates per block was high (~203–265 inmates) and we had limited resources. That investigation will imply time, logistics and financial resources. In addition, we did not evaluate prison staff, family members or visitors which could increase the number of TB cases detected and consequently increase the prevalence of active TB. Recently, one study reported that security guards may serve as a bridge population for TB transmission between prison and community [68].

In conclusion, TST positivity and active TB prevalence were higher than in the Colombian general population. Active TB case finding will increase the detection of new cases, we were able to detect 24 new cases in 3 weeks by active search finding. Detection, isolation and appropriate treatment of active TB cases would help to halt TB transmission in this prison. We found that TB patients from three clusters of two patients each with matching genotypes had epidemiological link. These findings suggest the need to implement an effective TB control program in prisons in order to prevent and reduce the TB prevalence, otherwise the public health problem of TB in these settings will remain and/or increase.

## Supporting information

S1 Questionnaire 1 and 2 Spanish version. (PDF)

S2 Questionnaire 1 and 2 English version. (PDF)

S1 Dataset (XLSX)
