# Supplementary material for: Active and latent tuberculosis among inmates in La Esperanza prison in Guaduas, Colombia
Source: PLoS One. 2019 Jan 25;14(1):e0209895. doi: 10.1371/journal.pone.0209895 (PMC6347203; doi:10.1371/journal.pone.0209895)
Supplement: S1 Questionnaire — (PDF) [file pone.0209895.s001.pdf]

(Supplementary file 1)

PROYECTO DE INVESTIGACION

Determinación de la Situación de la Tuberculosis en la población privada de la libertad interna en la cárcel La Esperanza de Guaduas (Cundinamarca).

CONFIDENCIALIDAD: Los datos suministrados a la Universidad Nacional de Colombia – Sede Bogotá, son confidenciales y no podrán utilizarse para fines comerciales, de tributación fiscal o investigación judicial.

ENCUESTA 1. DETECCIÓN DE SINTOMÁTICOS RESPIRATORIOS

Fecha: (Día) ☐☐ (Mes) ☐☐ (Año) ☐☐☐☐

Nombre y Apellidos del Encuestador:

1. Información general

**1.** Número del PACIENTE en el estudio:	
**2.** Nombres y Apellido(s) del Paciente:	
**3.** Documento de Identificación:	☐ (Sin identificación) ☐ (C.C.) ☐ (Cédula Extranjería)<br>Número de documento: ____________
**4.** Edad en años cumplidos:	☐☐
**5.** Numero de TD:	
**6.** Tipo de patio:	☐ Minima ☐ Mediana ☐ Maxima
**7.** Patio numero: _______	
**8.** De acuerdo con su cultura, pueblo o rasgo físico, ¿usted es o se reconoce como?	☐ Blanco. ☐ Indígena. ☐ Mestizo. ☐ Rom ☐ Raizal<br>☐ Palenquero. ☐ Negro, mulato, afrodescendiente. ☐ Otro, ¿Cuál? ____________
**9.** ¿Cuán tiempo lleva recluido en esta cárcel?	☐ Menos de 1 mes ¿Cuantos días?_______ ☐ 1 mes o más ¿Cuantos meses?_______
**10.** ¿Dentro de su condena actual, ha estado en otra cárcel?	☐ NO ☐ SI ¿Cuánto tiempo estuvo recluido en la otra carcel? (en meses): _______
**11.** ¿Antes de esta condena, usted había estado recluido o preso?	☐ NO ☐ SI *¿Cuántos tiempo estuvo recluido?*<br>☐ Menos de 1 mes, ¿Cuántos días? _____ días<br>☐ 1 mes o mas, ¿Cuántos meses? _____ meses
**12.** En qué ciudad o municipio vivía antes de estar recluido:	
**13.** ¿Cuál fue su último de año de estudio cursado?:____________	

14. ¿Qué ocupación tenía antes de estar recluido?

**Ocupación**	SI	NO
Trabajador del área de la salud (personal médico, de enfermería, bacteriología, fisioterapia, autopsias, patólogos, infectólogos y neumólogos)		
Trabajador en lugares encerrados o hacinados (cárceles, ejército, instituciones de largo plazo para el cuidado de los ancianos)		
Trabajo con minería, explotación de canteras, construcción de túneles o con muchos minerales metálicos *(Ver tarjeta anexa)*		
Trabajos expuestos a la inhalación de polvos –Asbesto- (Fabricación de tejidos, cartones, automóviles e instalaciones que contengan amianto)		
Trabajo con tóxicos orgánico como ácido, alcoholes, disolventes, pegante para zapatos.		
Otros trabajos, Cuáles:		
Ninguna ocupación		

15. Afiliación al Sistema de Seguridad Social en Salud (Marque con una X)

Régimen Contributivo	Régimen Subsidiado	No afiliado	Régimen Especial (Ejercito, Policía, Universidad Nacional, ECOPETROL, Magisterio)	Vinculado (SISBEN)

**16.** Nombre de la Entidad Administradora de Planes de Beneficios (EAPB) a la que pertenece. Para mayor facilidad preguntar por la EPS	☐ CAPRECOM ☐ ¿Otra? Cual_______________

2. Antecedentes clínicos de TB

**17.** ¿Ha presentado tos de cualquier duración recientemente?	NO<br>Pase a la 21	SI<br>Responda la 18, 19 y 20	¿Cuantos días seguidos lleva con la tos? ☐☐
**18.** ¿La tos está acompañada de flema, catarro o expectoración?	NO	SI	¿Cuantos días con la flema, catarro o expectoración? ☐☐
**19.** ¿Cuál es el color de la flema o espectoracion?<br>☐ Blanca ☐ Amarilla ☐ Verde	**20.** ¿La flema o expectoracion presentaba sangre?	SI	NO

**21.** ¿Usted tiene o ha tenido contacto con alguna persona diagnosticada con tuberculosis?	No sabe	NO	SI	***¿Quién fue esa persona?***<br>¿El contacto es o fue con un interno de la misma celda? SI NO<br>¿El contacto es o fue con un interno dentro de la cárcel? SI NO<br>¿El contacto es o fue con un visitante (familiar)? SI NO<br>¿El contacto es o fue con antes de estar recluido? SI NO

**22.** ¿Alguna vez ha sufrido de tuberculosis?	No sabe<br>Pase a la 24	NO<br>Pase a la 24	SI<br>Responda las preguntas 23
**23.** ¿Hace cuánto tiempo sufrió de tuberculosis? (en meses)	_____ meses	No sabe	No recuerda
**24.** ¿Ha tomado tratamiento para la tuberculosis en algún momento de su vida?	NO	SI	***¿Cuáles medicamentos tomo?***
**25.** ¿Con cuántas personas comparte su celda?	Numero	No sabe	
**26.** ¿Le han realizado la prueba del VIH/SIDA?	No sabe<br>Pase a la 29	NO<br>Pase a la 29	SI<br>Responda las preguntas 27 y 28
**27.** ¿Cuál fue el resultado de la prueba de VIH/SIDA?	No sabe	Neg	Pos
**28.** ¿Hace cuánto tiempo que le hicieron la prueba del VIH/SIDA? (en meses)	_____ meses		
**29.** ¿Actualmente está tomando tratamiento para VIH/SIDA?	No sabe<br>Pase a la 31	NO<br>Pase a la 31	SI<br>Responda la pregunta 30
**30.** ¿Ha interrumpido el tratamiento para VIH actual?	NO	SI	¿Hace cuánto lo interrumpió? *(mes/año):*
**31.** Diagnóstico de Sintomático respiratorio:	SI	NO	

3. Prueba de Tuberculina

(Si no se evidencian factores de riesgo y el interno lleva 3 meses o más recluido, se procederá a aplicarle la prueba. Para sacar el número de meses se suman las respuestas de las preguntas 9, 10 y 11). Si se evidencia VIH u otro factor de riesgo de inmunosupresión se aplica la prueba independiente del tiempo de reclusión del interno.

**A.** Fecha de realización *(día/mes/año)*:	Nombre y apellido del profesional:
**B.** Fecha de lectura final *(día/mes/año):*	Nombre y apellido de quien hizo la lectura final:

Resultado de la prueba: Diámetro (mm):_______

(Supplementary file 1)

PROYECTO DE INVESTIGACION

Determinar la Situación de la Tuberculosis en la población privada de la libertad interna en la cárcel La Esperanza de Guaduas (Cundinamarca).

CONFIDENCIALIDAD: Los datos suministrados a la Universidad Nacional de Colombia – Sede Bogotá, son confidenciales y no podrán utilizarse para fines comerciales, de tributación fiscal o investigación judicial.

ENCUESTA 2. EVALUACIÓN CLÍNICA DE LOS SINTOMÁTICOS RESPIRATORIOS

1. Identificación del interno	
Número del PACIENTE en el estudio:	Fecha de la evaluación clínica *(día/mes/ año)*:
Nombre completo del paciente:	Numero de TD:
**3.** Documento de Identificación:	☐ (Sin identificación) ☐ (C.C.) ☐ (Cédula Extranjería)<br>Número de documento: ____

2. Factores de riesgo para Tuberculosis		
**1.** ¿Le han realizado la prueba de la tuberculina?	No sabe / NO / SI	
**2.** ¿Ha tenido o tiene cáncer?	No sabe / NO / SI	Órgano / Tiempo de evolución (meses)
**3.** ¿Le han realizado algún trasplante?	No sabe / NO / SI	Órgano / Fecha *(día/mes/año)*
**4.** ¿Tiene diabetes Mellitus?	No sabe / NO / SI	Tipo I / Tipo II / Tiempo de evolución (meses)
**5.** ¿Ha tenido artritis reumatoide?	No sabe / NO / SI	Tiempo de evolución (meses)
**6.** ¿Ha tenido LUPUS?	No sabe / NO / SI	Tiempo de evolución (meses)
**7.** ¿Le han realizado una gastrectomía?	No sabe / NO / SI	Fecha *(día/mes/año)*
**8.** ¿Ha tenido o tiene Neumonía?	No sabe / NO / SI	
**9.** ¿Consume o ha consumido usted sustancias psicoactivas?	NO (Pase a la 10) / SI	***¿Cuáles consume?***<br>☐ Marihuana ☐ Heroína ☐ Bazuco<br>☐ Cocaína ☐ Pegantes ☐ Inhalantes<br>☐ Otras ¿Cuáles?:_______________________________<br>Tiempo de consumo (meses)_______________
**10.** ¿Fuma usted cigarrillo o tabaco? (definición de fumar como inhalar)	NO (Pase a la 12) / SI (Pase a la 11)	**11.** ¿Con que frecuencia fuma usted en la semana? ¿Cuántos cigarrillos fumó ayer _____ en la última semana _____
**12.** ¿Consume usted bebidas alcohólicas?	NO (Pase a la 14) / SI (Pase a la 13)	**13.** ¿Con que frecuencia consume bebidas alcohólicas en la semana?___________________
**14.** ¿En los últimos 6 meses ha tomado alguno de estos medicamentos?		Drogas para el cáncer: SI / NO<br>Antirretrovirales para el VIH: SI / NO<br>Prednisolona: SI / NO

3. Hallazgos clínicos

3.1 Síntomas relacionados con la presencia de TB y/o Micobacteriosis (marque con X)

**Síntomas**	**NO**	**SI**	**Tiempo de evolución (días)**	**Síntomas**	**NO**	**SI**	**Tiempo de evolución (días)**
Fiebre				Hemoptisis			
Tos				Dolor de cabeza			
Expectoración				Dolor abdominal			
Pérdida de peso				Diarrea			
Sudoración nocturna				Pérdida del apetito			

Adinamia y Astenia					Dolor torácico			
Hematuria					Escalofríos			

Otros síntomas respiratorios **¿Cuáles?**		Tiempo de evolución (días):
		Tiempo de evolución (días):
		Tiempo de evolución (días):

3.2 Examen físico

Cicatriz de BCG **(observe en el paciente)**	SI NO	Temperatura axilar **(En grados centígrados)**	
Peso **(kg)**		Tensión arterial	
Talla **(En centímetros)**		Frecuencia respiratoria **(Respiraciones / min)**	

Frecuencia cardiaca **(Latidos / min)**	

Signos de dificultad respiratoria (taquipnea, tirajes, cianosis, etc…)	NO	SI	**¿Cuáles?**
Agregados pulmonares (Roncus, sibilancias, estertores)	NO	SI	**¿Cuáles?**
Adenopatías (cervicales, inguinales, axilares, etc…)	NO	SI	**¿Cuáles?**

3.3 Diagnósticos clínicos

Nombre del médico:_________________________________ **Firma del médico**________________________
