# Supplementary material for: Active and latent tuberculosis among inmates in La Esperanza prison in Guaduas, Colombia
Source: PLoS One. 2019 Jan 25;14(1):e0209895. doi: 10.1371/journal.pone.0209895 (PMC6347203; doi:10.1371/journal.pone.0209895)
Supplement: S2 Questionnaire — (PDF) [file pone.0209895.s002.pdf]

(Supplementary file 2)

Research project

Determination of the Status of Tuberculosis in La Esperanza prison in Guaduas municipality, (Cundinamarca - Colombia).

CONFIDENTIALITY: The data provided to the National University of Colombia - Bogotá Headquarter, are confidential and may not be used for commercial purposes, taxation or judicial investigation.

Questionnaire 1. Detection of inmates with respiratory symptoms

Date: (Day) (Month) (Year)

Interviewer's complete name:

1. General information					
**1.** Inmate ID number in study:					
**2.** Inmate complete name and surname:					
**3.** Identification document:	(No ID)	(Colombian ID)	(ID of another country) ID number: ________		**4.** Age:
**5.** Jail ID number:	**6.** Securty Level: Minimum / Medium / Maximum			**7.** Block number: ______	
**8.** According to your culture, town or physical features, how do you recognize yourself?	White. / Indígena / Mestizo / Rom / Raizal / Palenquero. / Negro, mulato, afrodescendiente. / Other, ¿Which? ________				
**9.** ¿How long have you been in this jail?	Less than a month	¿number of days?______	1 moth or more	¿number of months?______	
**10.** Within his current sentence, have you been in another jail?	NO	yes	How long have you held in the other jail? (en meses): ______		
**11.** Before this imprisonment, have your ever been imprisoned?	NO	yes	***How long were you incarcerated?*** Less than a month, number of day? _____ days / 1 moth or more, number of months? _____ months		
**12.** In what city or municipality you lived before being incarcerated:		**13.** What was your last year of study completed?:________			
**14.** What occupation did you have before being incarcerated?	(see table below)				
**15.** Affiliation with the Social Security Health System (Mark with an X)	Contributive Regime	Subsidized Regime	Not affiliated	Special Regime (Army, Police, National University, ECOPETROL, Magisterio)	(SISBEN)

Occupation	YES	NO
Health worker (medical staff, nursing, bacteriology, physiotherapy, autopsies, pathologists, infectious diseases and pulmonologists)		
Worker in enclosed or crowded places (prisons, army, long-term institutions for the care of the elderly)		
Work with mining, exploitation of quarries, construction of tunnels or with many metallic minerals		
Work exposed to the inhalation of dust - Asbestos - (fabrics, cardboard, automobiles and installations containing asbestos)		
Work with toxic chemicals such as acid, alcohols, solvents, glue for shoes.		
Other works, Which:		
No occupation		

**16.** Name of the Administrative Entity of Benefit Plans to which it belongs. For more ease ask about EPS	☐ CAPRECOM ☐ Other? Which___

2. Clinical background of TB

**17.** Have you had cough of any duration recently?	NO<br>Go to 21	yes — How many days in a row does this cough take? ☐☐<br>Answer questions 18, 19 and 20
**18.** The cough is accompanied by phlegm, catarrh, or expectoration?	NO	yes — How many days do you have phlegm? catarrh, or expectoration? ☐☐
**19.** What is the color of phlegm or expectoration? ☐ White ☐ Yellow ☐ Green		**20.** Did the phlegm or expectoration have blood? NO yes

**21.** Do you have or have you had any contact with someone diagnosed with tuberculosis?	Does not know	NO	yes — ***Who was that person?***<br>Was the contact with an inmate in the same cell? YES NO<br>Is the contact or was it with an inmate inside the jail? YES NO<br>Is the contact or was it with a visitor (family member)? YES NO<br>Is the contact or was it before being incarcerated? YES NO

**22.** Have you ever suffered of tuberculosis?	Does not know<br>Go to 24	NO<br>Go to 24	yes<br>Answer question 23
**23.** How long have you suffered of tuberculosis? (in months)	______ months	Does not know	He does not remember
**24.** Have you taken treatment for tuberculosis at some time in your life?	NO	yes	***Which medications did you take?***
**25.** With how many people do you share your cell?	Number	Does not know	
**26.** Have you been tested for HIV/AIDS?	Does not know<br>Go to 29	NO<br>Go to 29	yes<br>Answer questions 27 and 28
**27.** What was the result of the HIV/AIDS test?	Does not know	Neg	Pos
**28.** How long have you been tested for HIV/AIDS? (in months)	______ months		
**29.** Are you currently taking treatment for HIV/AIDS?	Does not know<br>Go to 31	NO<br>Go to 31	yes<br>Answer question 30
**30.** Have you interrupted the current HIV treatment?	NO	yes — How long ago did you interrupt the treatment? (month year): ______ months	
**31.** Diagnosis of Respiratory Symptom:	YES	NO	

3. Tuberculin Skin TEST (TST)

(If there are no risk factors and the inmate has been incarcerated for 3 months or more, the test will be applied.) To get the number of months, add the answers to questions 9, 10 and 11). If there is evidence of HIV or another risk factor for immunosuppression, the test is applied to the inmate, regardless of the inmate's detention time.

**A.** TST application date *(day/month/year)*:	Name of the person who applied the test:
**B.** Date of reading the TST *(day/month/year)*:	Name of who did the reading test:
**TST result:** Diameter (mm):_______	

(Supplementary file 2)

Research project

Determination of the Status of Tuberculosis in La Esperanza prison in Guaduas municipality, (Cundinamarca - Colombia)

CONFIDENTIALITY: The data provided to the National University of Colombia - Bogotá Headquarter, are confidential and may not be used for commercial purposes, taxation or judicial investigation.

Questionnaire 2. Clinical evaluation of inmates with respiratory symptoms

**1. Inmate identification**				
Inmate ID number in study:			Date of clinical evaluation *(day/month/year)*:	
Inmate complete name:			Jail ID number::	
**3.** Identification document:	☐ (No ID)	☐ (Colombian ID)	☐ (ID of another country)	ID number: ___________________________

**2. Tuberculosis risk factor assesment**					
**1.** Have you been tested for tuberculin skin test?	Does not know	NO	yes		
**2.** Have you had or do you have cancer?	Does not know	NO	yes	Organ	Time of evolution (months)
**3.** Have you had any organ transplant?	Does not know	NO	yes	Organ	Date (day/month/year)
**4.** Do you have diabetes Mellitus?	Does not know	NO	yes	Type I / Type II	Time of evolution (months)
**5.** Have you had rheumatoid arthritis?	Does not know	NO	yes	Time of evolution (months)	
**6.** Have you had LUPUS?	Does not know	NO	yes	Time of evolution (months)	
**7.** Have you had a gastrectomy?	Does not know	NO	yes	Date (day/month/year)	
**8.** Have you had or do you have pneumonia?	Does not know	NO	yes		
**9.** Do you consume or have you used psychoactive substances?	NO<br>Go to 10	yes		***What drugs do you use?***<br>☐ Marihuana ☐ Heroine ☐ Bazuco<br>☐ Cocaine ☐ Pegantes ☐ Inhalants<br>☐ Other, Which? ______________________<br>Consumption time (months)______________	
**10.** Do you smoke cigarettes or tobacco? (definition of smoking as inhale)	NO<br>Go to 12	yes<br>Go to 11		**11.** How often do you smoke during the week? How many cigarettes did you smoke yesterday ______, in the last week ____	
**12.** Do you drink alcoholic beverages?	NO<br>Go to 14	yes<br>Go to 13		**13.** ¿ How often do you consume alcoholic beverages during the week?___________________	
**14.** In the last 6 months have you taken any of these medications?	Drugs for cancer?	yes	NO		
	Antiretroviral for HIV	yes	NO		
	Prednisolone	yes	NO		

3. Clinical findings

3.1 Symptoms related to the presence of TB and / or mycobacteriosis (check with X)

**Symptoms**	**NO**	**YES**	**Time of evolution (days)**	**Symptoms**	**NO**	**YES**	**Time of evolution (days)**
Fever				Hemoptysis			
Cough				Headache			
Expectoration				Abdominal pain			
Weight loss				Diarrhea			
Night sweats				Loss of appetite			

Adynamia and Asthenia					Chest pain			
Hematuria					Shaking chills			

Other respiratory symptoms		Time of evolution (days):
		Time of evolution (days):
		Time of evolution (days):

3.2 Physical exam

**BCG scar** (look at the patient)	yes NO	Axillary temperature (In degrees centigrade)	
**Weight** (kg)		Blood pressure	
Height (In centimeters)		Breathing frequency (breaths per minute)	
Heart rate (Beats / min)			

Signs of respiratory distress (tachypnea, runs, cyanosis, etc ...)	NO	yes	**Which?**
Pulmonary aggregates (Roncus, wheezing, rales)	NO	yes	**Which?**
Adenopathies (cervical, inguinal, axillary, etc ...)	NO	yes	**Which?**

3.3 Clinical diagnoses

Name of the doctor:________________________________ **Doctor's signature** _______________________
